# Profiling dendritic cell subsets in head and neck squamous cell tonsillar cancer and benign tonsils

**DOI:** 10.1038/s41598-018-26193-y

**Published:** 2018-05-23

**Authors:** Milad Abolhalaj, David Askmyr, Christina Alexandra Sakellariou, Kristina Lundberg, Lennart Greiff, Malin Lindstedt

**Affiliations:** 1 Department of Immunotechnology, Lund University, Lund, Sweden; 2 Department of ORL, Head & Neck Surgery, Skåne University Hospital, Lund, Sweden; 3 Department of Clinical Sciences, Lund University, Lund, Sweden

## Abstract

Dendritic cells (DCs) have a key role in orchestrating immune responses and are considered important targets for immunotherapy against cancer. In order to develop effective cancer vaccines, detailed knowledge of the micromilieu in cancer lesions is warranted. In this study, flow cytometry and human transcriptome arrays were used to characterize subsets of DCs in head and neck squamous cell tonsillar cancer and compare them to their counterparts in benign tonsils to evaluate subset-selective biomarkers associated with tonsillar cancer. We describe, for the first time, four subsets of DCs in tonsillar cancer: CD123^+^ plasmacytoid DCs (pDC), CD1c^+^, CD141^+^, and CD1c^−^CD141^−^ myeloid DCs (mDC). An increased frequency of DCs and an elevated mDC/pDC ratio were shown in malignant compared to benign tonsillar tissue. The microarray data demonstrates characteristics specific for tonsil cancer DC subsets, including expression of immunosuppressive molecules and lower expression levels of genes involved in development of effector immune responses in DCs in malignant tonsillar tissue, compared to their counterparts in benign tonsillar tissue. Finally, we present target candidates selectively expressed by different DC subsets in malignant tonsils and confirm expression of CD206/MRC1 and CD207/Langerin on CD1c^+^ DCs at protein level. This study descibes DC characteristics in the context of head and neck cancer and add valuable steps towards future DC-based therapies against tonsillar cancer.

## Introduction

Head and neck squamous cell cancer (HNSCC) comprises malignancies localized to the sinonasal cavities, oral cavity, pharynx, and larynx^1,2^. It is the sixth most frequent cancer with around 550,000 cases diagnosed and 300,000 lives lost worldwide per year^3^. A spectrum of different factors is known to cause HNSCC including smoking, alcohol abuse, high-risk types of human papillomavirus (HPV), and genetic factors^2^. A relatively high mortality rate and side effects from treatment, notably surgery and radio/chemotherapy, warrant a need for new strategies^4^. Among approaches addressing such a need, tumor-specific immunotherapy is promising given its targeting specificity^4^.

Dendritic cell (DC) targeting is a promising approach to treat cancer, considering DCs’ efficient capacity to internalize and process antigen, and initiate T cell responses through presentation via MHC molecules^5^. The main goal is to engineer a tumor-specific cytotoxic T lymphocyte (CTL) response which will eventually break the lesion’s immunosuppressive microenvironment and confer cancer cell eradication^6^. In DC-targeting approaches *in vivo*, tumor antigens may be coupled to monoclonal antibodies specific to surface markers and, upon delivery, DCs will present/cross-present the delivered antigens (Ag) via either MHC-II or MHC-I molecules resulting in antigen-specific T helper (Th) or CTL responses, respectively^7^.

Any surface molecule mediating endocytosis may then theoretically be considered as cargo vector, engaging an Ag in MHC-I or II presentation pathways. Some endocytic C-type lectin receptors (CLRs) such as CD205/DEC205 and CD207/Langerin may contribute to both MHC-I and II presentation^8,9^. Aside from endocytic surface molecules, triggering signaling molecules such CD40 and TLRs have likewise been shown to promote DCs priming capacities^10,11^. It was recently demonstrated in a murine vaccine model, delivering OVA Ag to five surface receptors, that neither expression levels, surface turnover, speed of Ag internalization, nor Ag load were major determinants for MHC I or MHC II presentation outcomes. Instead, it was the targeted receptors and the specializations of DC subsets that contributed to the overall outcome, an observation that remains to be confirmed with clinically relevant antigens^8^.

The plasticity of the DC subsets, their unique gene expression profiles and specialized functions, which depend on the local microenvironment, complicate targeting of DCs^12,13^. For instance, CD123^+^ plasmacytoid DCs (pDCs), CD1c^+^ myeloid DCs (mDCs), and CD141^+^ mDCs, three well-characterized DC subsets in benign tonsils^14^, show an overlapping level of functionality in terms of cross-presentation^15–17^. For example, on one hand, TLR9-activated pDCs have been shown to initiate a systemic immune response through triggering sequential activation of NK cells, mDCs, and CD8^+^ T cells upon direct administration in mouse melanoma tumors^18^ and, on the other hand, they have also been shown to contribute to tolerance development^19^. Similarly, mDCs have been shown to exhibit both tolerogenic and immunostimulatory properties depending on the prevailing micromilieu^20^. Thus, candidate targets that are DC subset specific with well-studied functional properties in the tumor environment are warranted to create desirable downstream immunological effects.

The aim of this study was to categorize DC subsets in tonsillar cancer, a HNSCC localized to the oropharynx, compared to benign tonsils, in order to outline immunological differences and to map out unique subset-specific expression of markers that may be potential targets in the development of future immunotherapeutics. We identified four DC subsets in tonsillar cancer through flow cytometry and compared the frequency of each tumor-resident subset to its counterpart in benign tonsils. We further took advantage of human transcriptome arrays to investigate gene expression in all the identified DC subsets, resulting in panels of surface markers selectively expressed by the individual DC subsets in malignant tonsillar tissue. Furthermore, we verified selective surface expression of CD206/MRC1 and CD207/Langerin on CD1c^+^ mDCs from malignant tonsillar tissue. The data sheds light on DC subsets’ characteristics in the cancer microenvironment and highlight markers that may be used for targeting specific DC subsets.

## Results

### Flow cytometric identification of DC subsets in tonsillar cancer and benign tonsils

**Figure 1:**
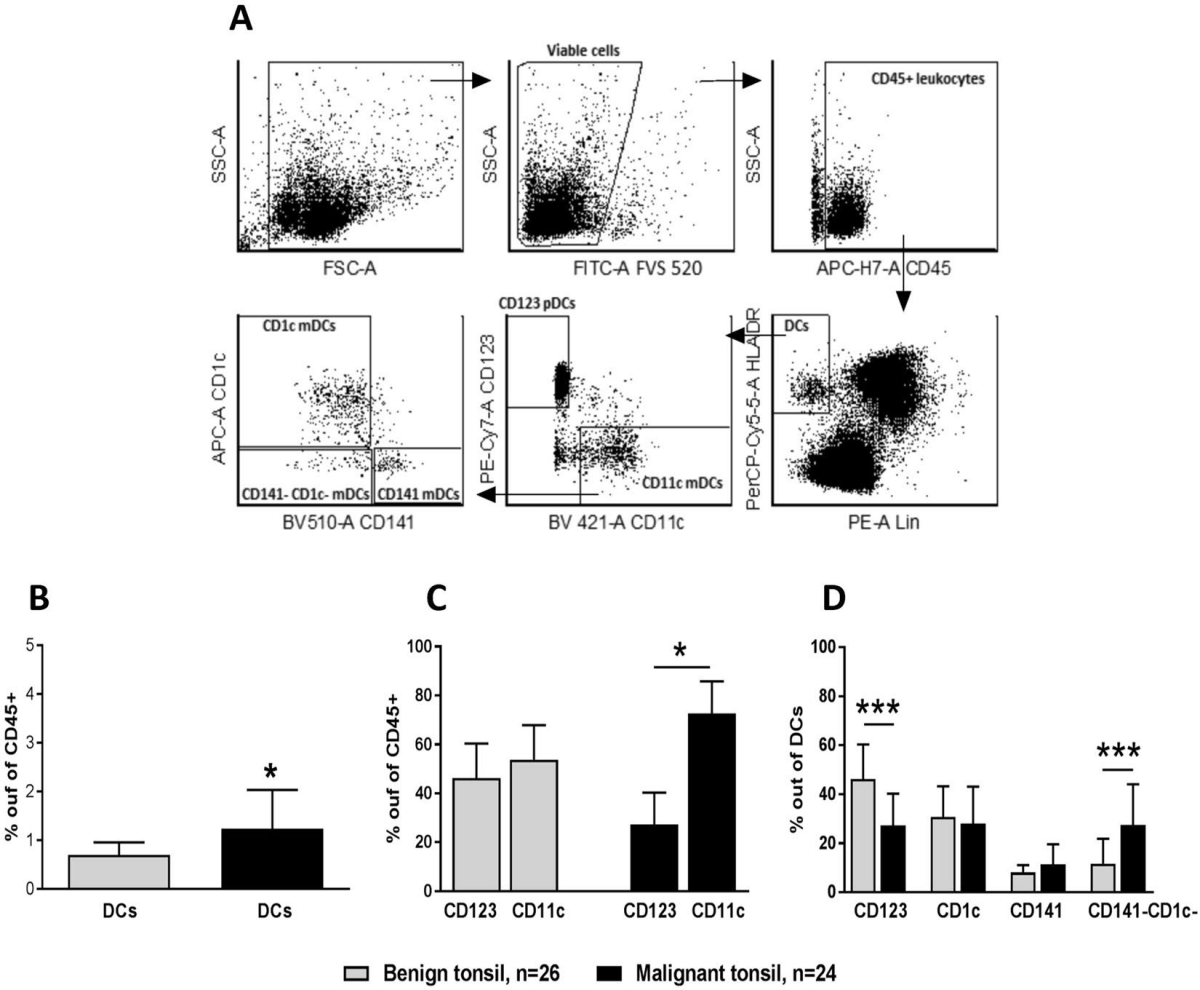
Sorting gate strategy and expression levels of DCs and DC subsets in malignant and benign tonsils. (**A**) Doublet exclusion was performed on viable cells (not shown). Leukocytes were then identified by expression of CD45, out of which, HLA-DR^+^ lineage^−^ (Lin) (CD3, CD14, CD56, and CD19 cells) cells were gated. Subsequently, mDCs and pDCs were identified based on expression of CD11c and CD123, respectively, forming the complete DC fraction. Myeloid DCs were further subdivided into CD141^+^, CD1c^+^, and DN cells based on expression of CD141, CD1c, or neither, respectively. (**B**) Frequencies of DCs among the present leukocytes in malignant tonsillar tissue compared to benign controls. (**C**) CD11c^+^ mDC/CD123^+^ pDC ratio in malignant and benign tonsils. (**D**) Frequency of CD123^+^ pDCs as well as DN mDCs in malignant and benign tonsils. *P < 0.05; ***P < 0.001.

Four DC subsets were identified in the lineage-negative and HLA-DR positive leukocyte populations of malignant and benign tonsillar tissue (Fig. 1A). CD11c+ myeloid cells were subdivided into CD1c^+^ , CD141^+^ and CD1c^−^ CD141^−^ DCs, and plasmacytoid DCs were identified using CD123. The percentage of DCs out of CD45^+^ leukocytes was significantly higher in tonsillar cancer (1.2 ± 0.8%) than in benign tonsils (0.7 ± 0.2%) (Fig. 1B). Furthermore, the ratio of CD11c^+^ mDCs/CD123^+^ pDCs was clearly elevated in malignant compared to benign tonsil tissue (Fig. 1C). Among the DC subsets, the percentage of CD123^+^ pDCs was significantly lower in tonsillar cancer (27.2 ± 13%) as compared to benign tonsils (46.2 ± 14.1%) (Fig. 1D). In contrast, the percentage of CD1c^−^CD141^−^ mDCs (double negative: DN) was significantly higher in malignant (27.4 ± 16.6%) compared to benign tonsillar tissue (11.7 ± 10.1%) (Fig. 1D). Levels of CD1c^+^ mDCs and CD141^+^ mDCs were similar between tonsillar cancer and benign tonsils (Fig. 1D).

### Subset transcriptome comparison in tonsillar cancer and benign tonsils

**Figure 2:**
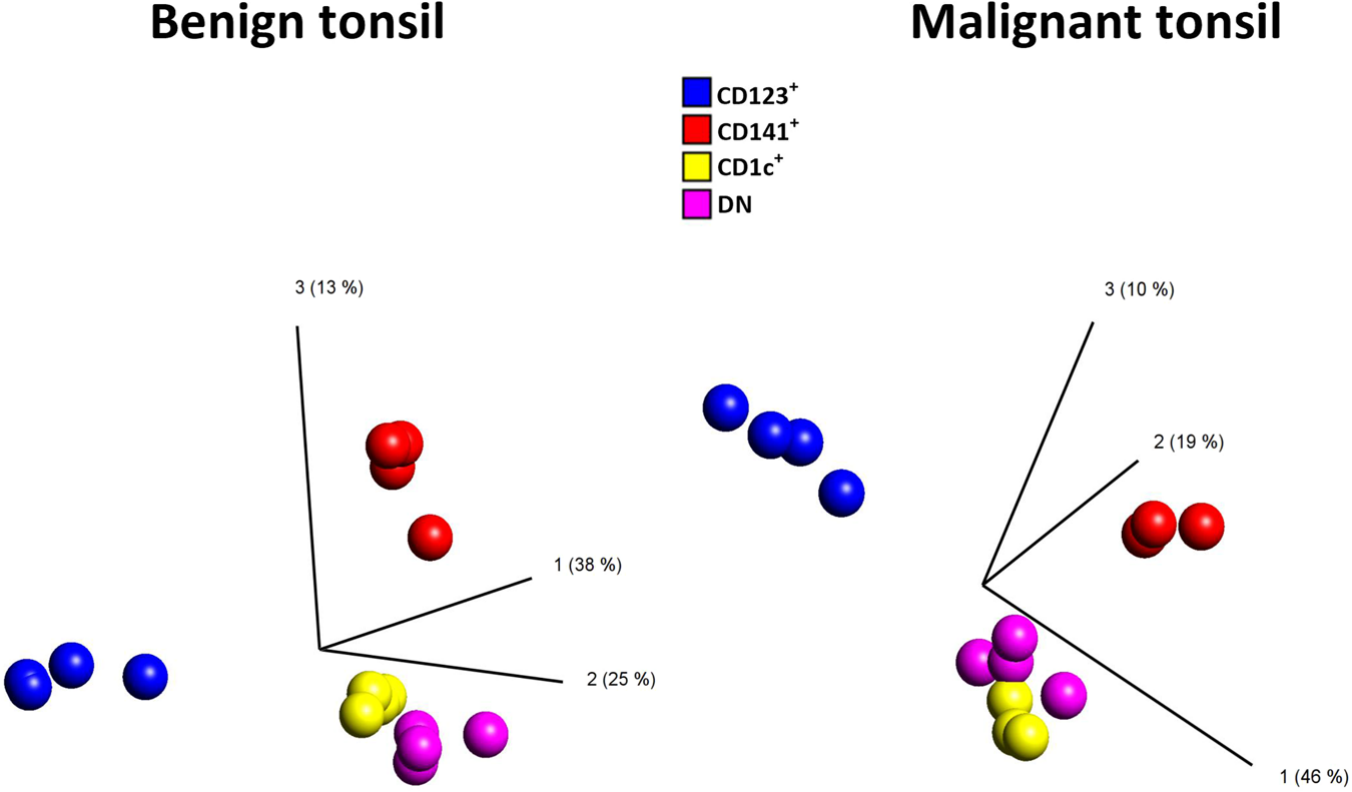
PCA of differentially expressed transcripts among DC subsets in benign and malignant tonsillar tissues. CD11c^+^ mDC subsets (CD1c^+^, CD141^+^, and DN) form a cluster separated from CD123^+^ pDC subset indicating higher transcriptional similarity among myeloid DCs.

DC subsets from malignant (n = 4) and benign tonsillar tissue (n = 4) were sorted to a purity exceeding 95% (data not shown) and RNA from each DC population was hybridized to Affymetrix Human Transcriptome Pico Assay 2.0 microarrays. Pools of 4,817 and 1,538 differentially expressed transcripts among the four subsets in benign and malignant tonsillar tissues, respectively, were identified by the filtering strategy. Principal component analysis (PCA) of the differentially expressed transcripts demonstrated separate clustering of the four subsets in benign as well as in malignant tonsillar tissues (Fig. 2). CD123^+^ pDCs were observed to cluster further away from the other three subsets, likely due to their different origin (Fig. 2A and B). Additionally, a lower number of differentially expressed transcripts observed in malignant tonsillar tissue indicated a higher degree of transcriptional similarity among DC subsets in tonsillar cancer as compared to benign tonsils, which may reflect that the tumor microenvironment has a strong influence on the expression profiles of the DC subsets.

### Characterization of DC subsets in tonsillar cancer

Pathway enrichment analysis was performed on the transcripts differentially expressed by DC subsets in malignant compared to benign tonsils, and the top ten pathway enrichment hits were selected (Tables 1–4) revealing mechanistic information linked to ongoing immune responses. For instance, the transcripts expressed at a higher level in CD1c^+^ mDCs in malignant compared to benign tonsils were enriched in pathways such as “Immune response- IL10 signaling pathway” and “Immune response- T cell co-signaling receptors” (including PD-L1, PD-L2, LAG3). CD123^+^ pDCs, CD141^+^ mDCs and the DN DCs in malignant tonsils display enriched transcripts in Interferon (IFN) α/β signalling via JAK/STAT pathway. Similarly, the “Immune response_Antiviral actions of Interferons” pathway was present in the higher expressed profiles of CD123^+^ mDCs and DN DCs. “PGE2 pathways in cancer” pathway was observed for the higher expressed profile of CD1c^+^ mDCs and the DN DC population.

**Table 1:** Top 10 pathway enrichment hits derived from differentially expressed transcripts in CD1c^+^ mDCs in malignant tonsil compared to CD1c^+^ mDCs in benign tonsils.

Enrichment hit	FDR	Ratio	Associated transcripts
**CD1c** ^**+**^ **mDCs malignant tonsils > CD1c** ^**+**^ **mDCs benign tonsils**			
Transcription_HIF-1 targets	6,649E-06	11/95	TGM2, MMP-9, GLUT3, Galectin-1, Adrenomedullin, Endoglin, PLGF, PLAUR (uPAR), F263, VEGF-A, NUR77
PGE2 pathways in cancer	9,204E-04	7/55	COX-2 (PTGS2), G-protein alpha-i family, EGR1, VEGF-A, Cyclin D1, TNF-alpha, PKA-reg (cAMP-dependent)
Immune response_IL-10 signaling pathway	1,391E-03	7/62	COX-2 (PTGS2), MMP-9, Bcl-XL, TIMP1, CD80, TNF-alpha, p19
Signal transduction_HTR2A signaling outside the nervous system	3,404E-03	7/74	COX-2 (PTGS2), G-protein alpha-i family, HB-EGF, EGR1, Calmodulin, HB-EGF(mature), Cyclin D1
Immune response_T cell co-signaling receptors, schema	3,970E-03	6/55	PD-L2, LAG3, Collagen XIII, PD-L1, CD80, Collagen III
Cell adhesion_ECM remodeling	3,970E-03	6/55	MMP-12, MMP-9, HB-EGF, PLAUR (uPAR), TIMP1, Collagen III
Development_Non-genomic action of Retinoic acid in cell differentiation	4,180E-03	6/57	RXRA, TGM2, COX-2 (PTGS2), PRKAR2B, VEGF-A, PKA-reg type II (cAMP-dependent)
Putative pathways of hormone action in neurofibromatosis type 1	1,230E-02	4/25	Adrenomedullin, EGR3, EGR1, EGR2 (Krox20)
Neurogenesis_NGF/ TrkA MAPK-mediated signaling	1,230E-02	7/105	HB-EGF, EGR1, PLAUR (uPAR), Calmodulin, NUR77, Cyclin D1, PKA-reg (cAMP-dependent)
Neurophysiological process_GABA-B receptor signaling at postsynaptic sides of synapses	1,230E-02	4/26	G-protein alpha-i family, G-protein alpha-o, ATF-5, Calmodulin
**CD1c** ^**+**^ **mDCs malignant tonsils < CD1c** ^**+**^ **mDCs benign tonsils**			
Signal transduction_Additional pathways of NF-kB activation (in the nucleus)	4,762E-08	12/30	p38alpha (MAPK14), I-kB, CBP, PRMT5, p90RSK1, STO, p300, PKA-cat alpha, PKC-delta, Adenylate cyclase, NFKBIA, IKK-beta
Immune response_Antigen presentation by MHC class II	2,948E-06	19/118	MHC class II alpha chain, HLA-DO, MHC class II, ARL14EP, FCGRT, ARHGEF2, PKC, HSP90, LLIR, MHC class II beta chain, PKC-alpha, PDK (PDPK1), PKC-delta, HCLS1, p38 MAPK, CLEC10A, A2M receptor, CD4, mTOR
Mucin expression in CF airways	7,533E-06	14/69	IL-1 beta, p38alpha (MAPK14), I-kB, c-Raf-1, p90RSK1, SP1, PLC-beta, MEKK1(MAP3K1), PKC-alpha, PKC-delta, p38 MAPK, PKA-cat (cAMP-dependent), p90Rsk, NFKBIA
Immune response_C5a signaling	7,533E-06	12/50	IL-1 beta, I-kB, c-Raf-1, Rac2, B-Raf, PKC, c-Fos, PLC-beta, PDK (PDPK1), p38 MAPK, p90Rsk, PREX1
Development_Glucocorticoid receptor signaling	7,533E-06	9/25	CBP, GCR Alpha, NCOA1 (SRC1), GCR Beta, Oct-2, HSP90, GCR, p300, NFKBIA
Signal transduction_Additional pathways of NF-kB activation (in the cytoplasm)	9,905E-06	12/52	I-kB, c-Raf-1, p90RSK1, MEKK1(MAP3K1), PKC-alpha, PDK (PDPK1), PKA-cat alpha, PKC-delta, Adenylate cyclase, NFKBIA, IKK-beta, Casein kinase II, alpha chain (CSNK2A1)
Immune response_IL-4 signaling pathway	3,280E-05	15/94	p38alpha (MAPK14), Tuberin, c-Raf-1, p90RSK1, c-Fes, PKC, IL13RA1, MEKK1(MAP3K1), PDK (PDPK1), Tyk2, PKC-delta, JAK3, p38 MAPK, NFKBIA, mTOR
Oxidative stress_Activation of NADPH oxidase	3,280E-05	12/59	TRIO, cPKC (conventional), Rac2, PKC, PLC-beta, PKC-alpha, PDK (PDPK1), PKC-delta, p114-RhoGEF, p38 MAPK, VAV-1, PREX1
Immune response_IL-6 signaling pathway via JAK/STAT	4,067E-05	13/72	CBP, Rac2, c-Fos, SP1, p300, sIL6-RA, MEKK1(MAP3K1), Tyk2, PKC-delta, JAK3, VAV-1, mTOR, IL6RA
Development_TGF-beta receptor signaling	5,370E-05	11/52	CBP, c-Raf-1, SP1, p300, SMAD2, TIEG1, YY1, TGF-beta receptor type II, p38 MAPK, NFKBIA, IKK-beta

**Table 2:** Top 10 pathway enrichment hits derived from differentially expressed transcripts in CD123^+^ pDCs in malignant tonsil compared to CD123^+^ pDCs in benign tonsils.

Enrichment hit	FDR	Ratio	Associated transcripts
**CD123** ^**+**^ **pDCs malignant tonsil > CD123** ^**+**^ **pDCs benign tonsils**			
Immune response_IFN-alpha/beta signaling via JAK/STAT	9,226E-10	9/64	IFI27, USP18, PKR, IFI17, IFI6, Apo-2L(TNFSF10), XAF1, STAT1, PML
Immune response_Antiviral actions of Interferons	2,333E-04	5/52	PKR, OAS2, 2′-5′-oligoadenylate synthetase, STAT1, MxA
Development_Role of CNTF and LIF in regulation of oligodendrocyte development	1,313E-02	3/28	Annexin V, STAT1, CLIC4
Immune response_IFN-alpha/beta signaling via MAPKs	1,415E-02	4/77	PKR, Apo-2L(TNFSF10), STAT1, PML
Macrophage and dendritic cell phenotype shift in cancer	3,050E-02	4/100	Apo-2L(TNFSF10), PGE2R4, STAT1, CD40(TNFRSF5)
Mitochondrial dysfunction in neurodegenerative diseases*	5,930E-02	3/59	G3P2, mGluR5, PPIF
Immune response_IL-4-responsive genes in type 2 immunity*	8,279E-02	3/70	STAT1, CD40(TNFRSF5), CCL17
SLE genetic marker-specific pathways in antigen-presenting cells (APC)*	1,080E-01	3/84	MDA-5, STAT1, CD40(TNFRSF5)
Immune response_Innate immune response to RNA viral infection*	1,080E-01	2/28	MDA-5, LGP2
Glycolysis and gluconeogenesis*	1,080E-01	3/94	G3P2, TPI1, GLUT3
**CD123** ^**+**^ **pDCs malignant tonsil < CD123** ^**+**^ **pDCs benign tonsils**			
Immune response_IL-4 signaling pathway	6,228E-04	15/94	Syk, IKK-gamma, Tuberin, Shc, STAT5, c-Fes, PKC, IL4RA, c-Cbl, MEKK1(MAP3K1), PDK (PDPK1), Tyk2, PKC-delta, NFKBIA, PI3K cat class IA (p110-delta)
Signal transduction_Additional pathways of NF-kB activation (in the cytoplasm)	6,228E-04	11/52	IKK-gamma, I-kB, PI3K cat class IA, Pin1, NIK(MAP3K14), MEKK1(MAP3K1), PDK (PDPK1), PKC-delta, Adenylate cyclase, NFKBIA, IKK-beta
Transcription_Negative regulation of HIF1A function	1,243E-03	12/69	ING4, MTG16 (CBFA2T3), Casein kinase I delta, KLF2, HIF-prolyl hydroxylase, EGLN2, MCM5, Sirtuin2, CHIP, CITED2, VHL, Sirtuin7
Signal transduction_Additional pathways of NF-kB activation (in the nucleus)	1,349E-03	8/30	IKK-gamma, I-kB, CBP, NIK(MAP3K14), PKC-delta, Adenylate cyclase, NFKBIA, IKK-beta
Signal transduction_PTMs (phosphorylation) in TNF-alpha-induced NF-kB signaling	1,629E-03	9/41	IKK-gamma, PI3K cat class IA, Pin1, NIK(MAP3K14), PDK (PDPK1), PKC-delta, Adenylate cyclase, NFKBIA, IKK-beta
Immune response_Gastrin in inflammatory response	3,646E-03	11/69	ELAVL1 (HuR), I-kB, Shc, PI3K cat class IA, MEF2, NIK(MAP3K14), MEKK1(MAP3K1), PDK (PDPK1), PKC-delta, MEF2B, IKK-beta
Immune response_IL-2 activation and signaling pathway	3,714E-03	9/48	Syk, HMGI/Y, I-kB, Shc, STAT5, PI3K cat class IA, Calcineurin A (catalytic), SMAD3, PDK (PDPK1)
Immune response_TREM1 signaling pathway	3,714E-03	10/60	Syk, IKK-gamma, I-kB, Bax, PI3K cat class IA, Calcineurin A (catalytic), c-Cbl, MIP-1-alpha, PDK (PDPK1), NFKBIA
Immune response_LTBR1 signaling	3,714E-03	10/60	Syk, I-kB, PI3K cat class IA, PKC, PLC-beta, PDK (PDPK1), PKC-delta, Adenylate cyclase, p47-phox, PLC-beta2
Signal transduction_IP3 signaling	3,714E-03	9/49	Syk, Shc, Galpha(q)-specific nucleotide-like GPCRs, PI3K cat class IA, MEF2, HDAC5, PLC-beta, PDK (PDPK1), CaMKK

**Table 3:** Top 10 pathway enrichment hits derived from differentially expressed transcripts in CD141^+^ mDCs in malignant tonsil compared to CD141^+^ mDCs in benign tonsils.

Enrichment hit	FDR	Ratio	Associated transcripts
**CD141** ^**+**^ **mDCs malignant tonsil > CD141** ^**+**^ **mDCs benign tonsils**			
Immune response_IFN-alpha/beta signaling via JAK/STAT	6,856E-04	5/64	IFI27, IP10, IRF1, GBP1, PML
Immune response_T regulatory cell-mediated modulation of antigen-presenting cell functions*	1,659E-01	3/66	IRF1, SLC7A5, LAG3
Cell cycle_Role of 14-3-3 proteins in cell cycle regulation*	1,717E-01	2/22	CDC25B, 14-3-3 sigma
NETosis in SLE*	1,987E-01	2/31	Histone H2, Histone H2A
Cell cycle_Spindle assembly and chromosome separation*	1,987E-01	2/33	TPX2, Aurora-B
Cell cycle_The metaphase checkpoint*	1,987E-01	2/36	SPBC24, Aurora-B
Apoptosis and survival_NGF activation of NF-kB*	1,987E-01	2/40	Bcl-XL, TGM2
Apoptosis and survival_BAD phosphorylation*	1,987E-01	2/42	Bcl-XL, 14-3-3
Immune response_CRTH2 signaling in Th2 cells*	1,987E-01	2/44	Bcl-XL, 14-3-3
Development_PIP3 signaling in cardiac myocytes*	1,987E-01	2/47	Bcl-XL, 14-3-3
**CD141** ^**+**^ **mDCs malignant tonsil < CD141** ^**+**^ **mDCs benign tonsils**			
Immune response_IL-11 signaling pathway via MEK/ERK and PI3K/AKT cascades	2,205E-04	9/67	I-kB, SFK, Osteocalcin, Fyn, gp130, NFKBIA, c-Jun, Caspase-9, c-Fos
Immune response_IL-6-induced acute-phase response in hepatocytes	2,205E-04	7/36	Heme oxygenase 1, c-Jun/c-Fos, gp130, IL-6 receptor, c-Jun, IL6RA, c-Fos
Reproduction_Gonadotropin-releasing hormone (GnRH) signaling	2,205E-04	9/72	c-Jun/c-Fos, AP-1, PER1, Adenylate cyclase, c-Jun, MKP-1, c-Fos, G-protein alpha-q/11, PLC-beta
Immune response_IL-6 signaling pathway via JAK/STAT	2,205E-04	9/72	sIL6-RA, Osteocalcin, c-Jun/c-Fos, gp130, AP-1, IL-6 receptor, c-Jun, IL6RA, c-Fos
Aberrant production of IL-2 and IL-17 in SLE T cells	3,228E-04	8/58	Syk, I-kB, Fc epsilon RI gamma, c-Jun/c-Fos, MHC class II, AP-1, CD4, c-Fos
Immune response_M-CSF-receptor signaling pathway	4,003E-04	9/81	Syk, GAB3, c-Jun/c-Fos, GLUT1, Fyn, AP-1, PLD2, c-Jun, c-Fos
Immune response_MIF-induced cell adhesion, migration and angiogenesis	5,059E-04	7/46	CD44, c-Jun/c-Fos, AP-1, CXCR4, c-Jun, MKP-1, c-Fos
Immune response_Histamine H1 receptor signaling in immune response	5,135E-04	7/47	I-kB, c-Jun/c-Fos, NFKBIA, c-Jun, c-Fos, G-protein alpha-q/11, PLC-beta
Immune response_IL-22 signaling pathway	1,089E-03	6/36	IL10RB, c-Jun/c-Fos, MHC class II, c-Jun, CD4, c-Fos
Development_Angiotensin signaling via PYK2	3,207E-03	6/44	c-Jun/c-Fos, AP-1, c-Jun, c-Fos, G-protein alpha-q/11, PLC-beta

**Table 4:** Top 10 pathway enrichment hits derived from differentially expressed transcripts in DN mDCs in malignant tonsil compared to DN mDCs in benign tonsils.

Enrichment hit	FDR	Ratio	Associated transcripts
**DN mDCs malignant tonsil > DN mDCs benign tonsils**			
Immune response_IFN-alpha/beta signaling via JAK/STAT	9,200E-06	9/64	p21, IRF1, IFI17, IFI6, Apo-2L(TNFSF10), MIG, STAT1, PML, GBP4
Immune response_Antiviral actions of Interferons	2,566E-04	7/52	IRF1, WARS, OAS2, HLA-A, 2′-5′-oligoadenylate synthetase, IDO1, STAT1
PGE2 pathways in cancer	3,632E-03	6/55	COX-2 (PTGS2), G-protein alpha-i family, NURR1, EGR1, COX-1 (PTGS1), Cyclin D1
Development_Thrombopoetin signaling via JAK-STAT pathway	8,911E-03	4/22	p21, Bcl-XL, STAT1, Cyclin D1
Role of IL-23/ T17 pathogenic axis in psoriasis	2,395E-02	5/54	IL-15RA, NF-AT1(NFATC2), HLA-Cw6, HLA-C, sIL-15RA
Cell cycle_Role of Nek in cell cycle regulation	2,694E-02	4/32	Tubulin beta, Tubulin alpha, Histone H1, Tubulin (in microtubules)
IGF family signaling in colorectal cancer	2,810E-02	5/60	COX-2 (PTGS2), Bcl-XL, Apo-2L(TNFSF10), Cyclin D1, Clusterin
Immune response_IL-6 signaling pathway via JAK/STAT*	5,703E-02	5/72	p21, IRF1, COX-2 (PTGS2), Rac2, STAT1
Immune response_IFN-alpha/beta signaling via MAPKs*	6,293E-02	5/77	p21, Apo-2L(TNFSF10), STAT1, PML, Cyclin D1
Immune response_IL-15 signaling via JAK-STAT cascade*	6,293E-02	3/22	IL-15RA, Bcl-XL, sIL-15RA
**DN mDCs malignant tonsil < DN mDCs benign tonsils**			
Transcription_Role of heterochromatin protein 1 (HP1) family in transcriptional silencing	3,951E-03	5/40	HP1, DNMT3A, HP1 beta, Mi-2, Mi-2 alpha
Development_PDGF signaling via MAPK cascades	4,431E-03	5/47	PDGF receptor, MEK1(MAP2K1), SRF, PKC-alpha, PDGF-R-beta
HBV signaling via protein kinases leading to HCC	1,353E-02	4/36	MEK1(MAP2K1), PKC-alpha, PKC, cPKC (conventional)
Development_Gastrin in differentiation of the gastric mucosa	1,353E-02	4/38	MEK1(MAP2K1), PKC-alpha, PKC, cPKC (conventional)
Nociception_Nociceptin receptor signaling	1,353E-02	5/76	Nociceptin receptor, PKC-alpha, MEK1/2, PKC, cPKC (conventional)
Oxidative stress_Activation of NOX1, NOX5, DUOX1 and DUOX2 NADPH Oxidases	1,353E-02	4/42	PKC-alpha, MEK1/2, PKC, cPKC (conventional)
Development_VEGF signaling and activation	1,353E-02	4/42	MEK1(MAP2K1), PKC-alpha, IKK-beta, PKC
Apoptosis and survival_Anti-apoptotic action of Gastrin	1,353E-02	4/43	MEK1(MAP2K1), SRF, PKC-alpha, Bax
Neurophysiological process_Dopamine D2 receptor transactivation of PDGFR in CNS	3,398E-02	3/26	PDGF receptor, PKC, PDGF-R-beta
Oxidative stress_Activation of NADPH oxidase	3,398E-02	4/59	PKC-alpha, MEK1/2, PKC, cPKC (conventional)

### Subset selective gene expression and confirmation of CD206 and CD207 surface expression

**Figure 3:**
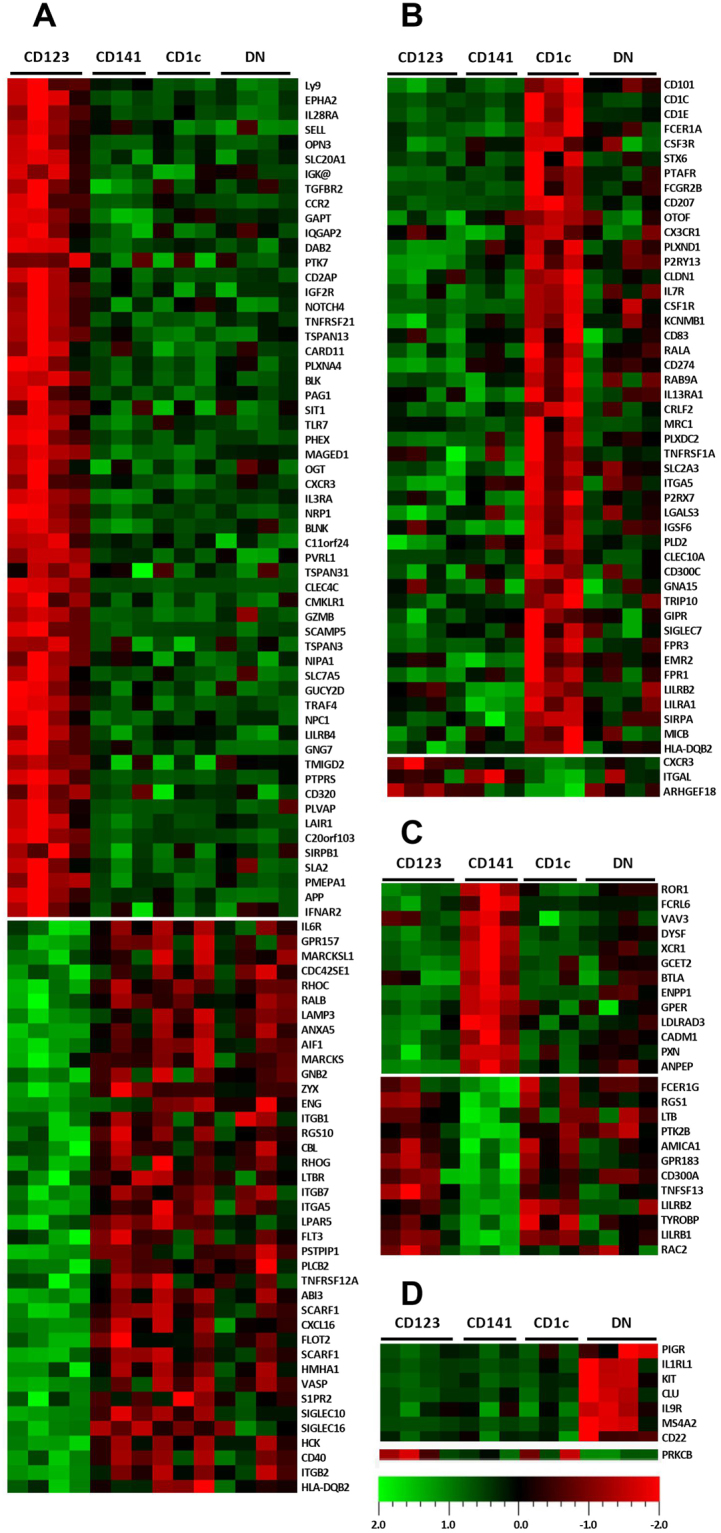
Candidate targeting molecules differentially expressed by CD123^+^ pDCs (**A**), CD1c^+^ (**B**), CD141^+^ (**C**), and DN mDCs (**D**), generated by reviewing the transcripts enriched in MetaCore “Plasma membrane” and “Cell surface” localization enrichments associated with endocytosis, cross presentation, or signaling activities. Transcripts were identified through one-tailed T-tests (p < 0.05, fold change ≥ 1.2) comparing each subset to other subsets in malignant tonsils separately.

**Figure 4:**
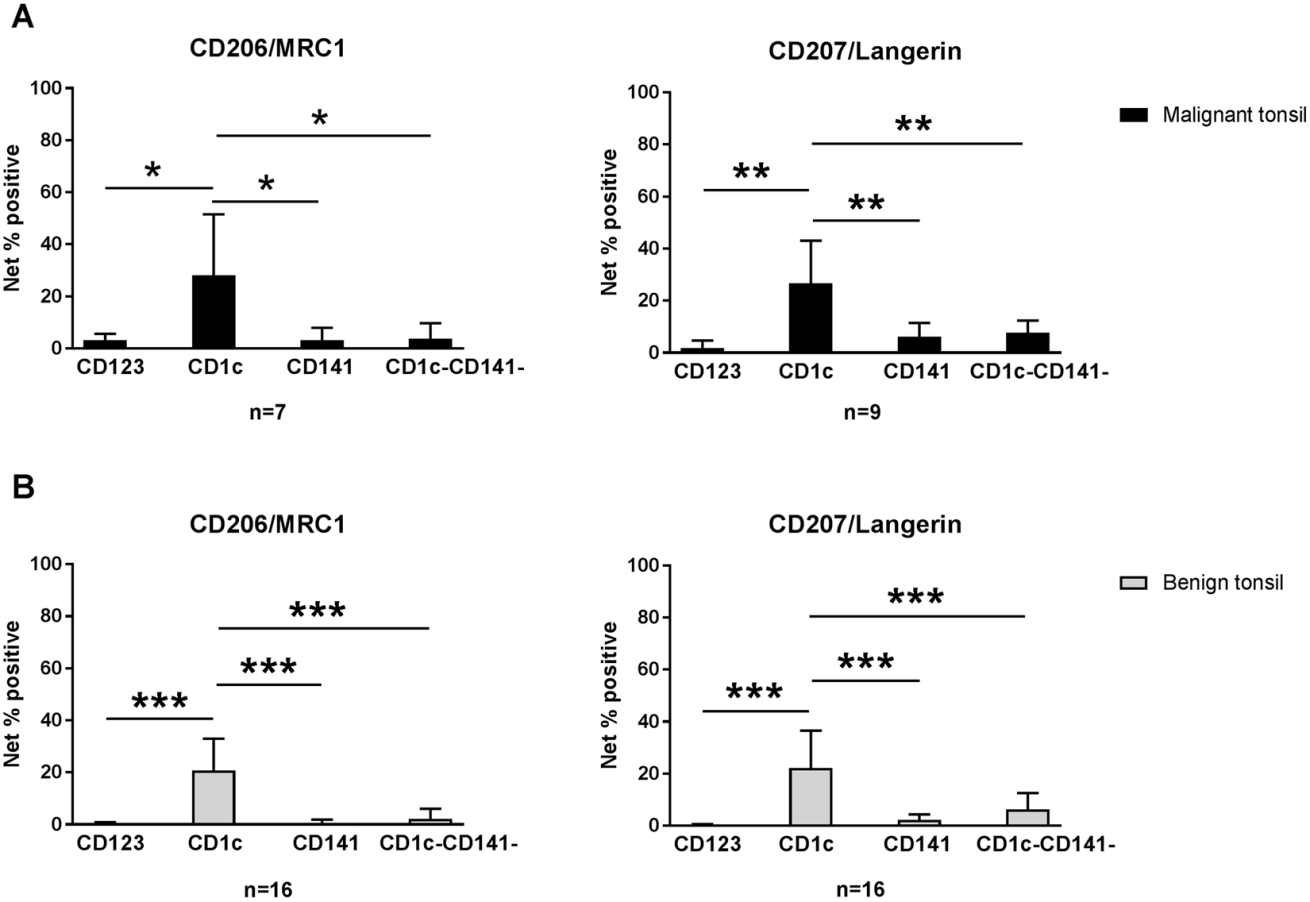
Expression of CD206/MRC1 and CD207/Langerin by CD1c^+^ mDCs in benign and malignant tonsils. Higher frequency of CD1c^+^ DCs express CD206/MRC1 and CD207/Langerin in malignant (**A**) and benign (**B**) tonsillar samples as compared to other subsets, measured by flow cytometry. Net percentage of positive cells was calculated by FMO positivity subtraction. *P < 0.05; **P < 0.01; ***P < 0.001.

Plasma membrane and cell surface associated transcripts that were selectively expressed by individual subsets within malignant tonsils are visualized in heatmaps (Fig. 3). A variety of molecules including CLRs, TLRs, adhesion molecules, receptors, inhibitory molecules, enzymes, and transporters were identified, representing candidate molecules for selective targeting of DC subsets. Given the small sample sizes of malignant biopsies available and the complex multicolor staining for flow cytometry, a wider phenotypic analysis was not technically feasible, yet selective surface expression of CD206/MRC1 and CD207/Langerin by the CD1c^+^ mDC subset was confirmed in malignant and benign tonsillar tissues (Fig. 4).

## Discussion

DCs regulate both humoral and cellular functions of the immune system and are therefore valuable targets for therapeutic purposes. In a context of cancer, clonal expansion of CTLs is warranted, which can be facilitated by instructions to specific DC subsets to cross-present antigen to T-cells. The purpose of this study is to provide a descriptive account on DC subsets’ characteristics in the tonsillar tumor microenvironment. Accordingly, we describe, for the first time, CD123^+^ pDCs, CD1c^+^ mDCs, CD141^+^ mDCs, and DN mDCs in tonsillar cancer and compare their transcriptional profiles to each other and to their counterparts in benign tonsils. The results are of relevance to attempts to develop DC-targeting immunotherapies for tonsillar cancer.

In this study, flow cytometry analysis demonstrated that CD123^+^ pDCs as well as CD1c^+^ and CD141^+^ mDCs, previously shown to be present in benign tonsillar tissue^14^, are also present in malignant tonsillar tissue. In addition, an HLA-DR^+^ Lin^−^ CD11c^+^ DC subset that lacks expression of CD1c and CD141 was identified, here referred to as DN mDCs. Attention should be paid to the fact that the CD1c^+^ mDCs in tissue may constitute a rather heterogeneic subset including migratory DCs, resident DCs and perhaps also inflammatory DCs derived from monocytes, and these are not further distinguished in this data set^21^. An overall higher frequency of DCs, out of CD45^+^ leukocytes, was observed in malignant as compared to benign tissue, suggesting that DC targeting may be feasible in tonsillar cancer. In addition, CD123^+^ pDCs were less frequent in malignant tissue, which could be advantageous to immunotherapy considering the role of this subset in tolerance development^19,22^. In contrast to CD123^+^ pDCs, the level of DN mDCs was higher in malignant as compared to benign tonsils. A similar DN mDC population, with dendritic cell-resembling profile, has been recently identified in human blood^23^. The herein presented data show that DNs in malignant tonsils express high levels of FTL, SERPINA1, LST1, AIF1, IFITM3, LILRB2, CSF1R, IFITM2 and PECAM1, in line with the demonstrated expression profile of the similar DC population identified in peripheral blood^23^. Further investigations are warranted to understand the origin and functional role of DN DCs.

Selective expression of several chemokine receptors was identified by our transcriptome profiling of the subsets in malignant tonsil, suggesting possible mechanisms for DCs to infiltrate the malignant tonsils. For example, CD123^+^ pDCs were found to selectively express CCR2, CMKLR1, and CXCR3, receptors known to be involved in DC maturation, trafficking and recruitment at tumor sites^24–26^ whereas XCR1 and CX3CR1, were selectively expressed by CD141^+^ mDCs and CD1c^+^ mDCs.

The selective expression of some inhibitory molecules by specific DC subsets from tonsillar cancer tissue suggests that these subsets may have an immunosuppressive profile. For instance, ILT4 (LILRB2), CD101, and PD-L1 (CD274) were shown to be selectively expressed by CD1c^+^ mDCs and BTLA by CD141^+^ mDCs. BTLA engagement on DCs has been reported to actively tolerize T cell responses^27^. Additionally, CD101/ILT4 duet on CD1c^+^ mDCs may have a critical role in diminishing T cell responses, as triggering CD101 results in IL-10 production that selectively upregulates ILT4, leading to T cell hypo-responsiveness^28,29^. In further support of an immunosuppressive profile, the IL-10 signaling pathway was an enrichment hit for CD1c^+^ mDCs in tonsillar cancer. Furthermore, a higher expression level of PD-L1, PD-L2, and LAG3 in CD1c^+^ mDCs from malignant compared to benign tonsils, as the corresponding pathway analysis suggests, may be an additional reflection of CD1c^+^ mDC suppression, as a role of PD-L1^+^ DCs in induction of regulatory T cell and tolerance has been shown^30^.

In this study, higher expression of angiogenesis related transcripts in the malignant CD1c^+^ mDC subset, such as VEGF-A, involved in the “Transcription_ HIF-1 targets” and in the “PGE2 pathway in cancer” pathways, may provide a further indication of immunosuppression produced by this particular DC subset. It has previously been reported in an *in vivo* ovarian cancer model that tumor infiltrating DCs have the ability to undergo endothelial-like differentiation, promoting tumor vasculogenesis, due to VEGF-A overexpression by DCs^31^. Further, *in vivo* studies of a breast cancer model have shown that a combinatorial treatment involving HIF-1 inhibitors, reducing the VEGF-A and FoxP3 expression, and a DC-based vaccine, gave rise to a strong anti-tumor cytotoxic T cell immune response with an increased IFN-γ production and reduced Treg cell activation^32^.

Despite observing a higher expression of immunosuppressive molecules by specific DC subsets in malignant compared to benign tonsillar tissue, it is important to highlight the higher expression of transcripts by CD123^+^ pDCs, CD141^+^ mDCs and the DNs, associated with IFN α/β signalling pathways, which may be linked to antiviral activity of these subsets towards HPV. It is well known that pDCs and mDCs produce Type I IFNs in response to viral infection^33^ and treating tumors with IFNs has shown some promise^34^. Moreover, many of the transcripts expressed at lower levels in the DC subsets in malignant tonsillar tissue are mostly enriched in pathways related to development of an effective immune response. For instance, “Immune response. Antigen presentation by MHC class II” was enriched by transcripts with lower expression level in CD1c^+^ DCs in malignant tissue. Interestingly, diminished Th1 response has been shown in patients with advanced HNSCC as compared to less advanced stages^35^. Also, complement C5a signaling was found to be expressed at lower levels, which may indicate a lower presentation capability of this subset^36,37^. Another interesting finding concerns IL4, as several transcripts involved in IL4 signaling were expressed to a lower degree in malignant tissue by CD1c^+^ DCs and CD123^+^ pDCs compared to those in benign tissue. Previous studies have shown that IL4 can abrogate DC suppression in the tumor microenvironment^38^, as well as activate tumor-infiltration of DCs, in a STAT-6 dependent manner, to promote type-1 T cell responses^39^. These observations suggest an underlining immunosuppressive environment based on inhibitory molecules and a lower expression of genes involved in development of effector immune responses. Further, IFN related observations may describe an insufficient immune anti-tumor/viral response in the microenvironment.

This study identified selectively expressed surface markers on specific DC subsets in tonsillar cancer, and CD206/MRC1 and CD207/Langerin on CD1c^+^ mDCs were confirmed at protein level. Targeting these particular receptors may efficiently induce cross-presentation of antigen and activation of CD4^+^ and CD8^+^ T cells^9,40^. Furthermore, out of the surface markers selectively expressed by CD141^+^ DCs, XCR1 is considered a promising target for antibody-based DC vaccines^41^. As examples of markers expressed by CD123^+^ DCs, targeting EPHA2 may produce anti-tumor effects^42,43^, while blocking of IL28RA result in augmented vaccine responses^44^. Information on DNs is lacking in the context of immunotherapy, but our analysis on their selective markers introduces candidates that, following functional studies, may emerge as immunotherapy targets. Taken together, selectively expressed molecules presented from this study may be considered as potential targets in future immunotherapy strategies^9,45,46^. Other receptors such as CLEC4a, DEC-205, and DC-SIGN, although not exclusively expressed on specific DC subsets, may still represent attractive targets^41,47,48^, depending on therapeutic strategy. Further studies are warranted to assess candidate marker protein expression and their efficacy in priming immune responses in tonsillar cancer.

In conclusion, this study describes four DC subsets in tonsillar cancer and their selective expression of surface targets. Our observations suggest overall immunosuppressive DC features, based on expression of inhibitory molecules and as well as of low expression of genes involved in development of immune effector responses, but observations on IFN may nevertheless suggest an existing yet insufficient immune response in tonsillar cancer.

## Materials and Methods

### Patients and sample preparation

Biopsies from tonsillar cancer lesions were obtained at Skåne University Hospital (Lund, Sweden). Benign tonsils were obtained from patients with tonsillar hypertrophy or recurrent tonsillitis (without signs of ongoing infection) undergoing tonsillectomy at Ängelholm and Helsingborg Hospitals (Sweden). It is confirmed that all research was performed in accordance with relevant guidelines/regulations, approved by the Regional Ethics Committee (EPN-Regionala Etikprövningsnämnden i Lund) and informed consent was obtained from all participants and/or their legal guardians. The tonsillar cancers were all tested positive for p16 expression, suggesting an association with human papilloma virus (HPV). The biopsies were cut into small pieces in RPMI 1640 medium (Thermo Fisher Scientific, Bremen, Germany) supplemented with 0.1 mg/ml gentamycin (Sigma Aldrich, St Louis, MO). Enzymatic digestion was then performed by incubating the tissue suspension at 37 °C for 20 minutes with Collagenase IV (Sigma Aldrich) (2 mg/ml) and DNase I (Sigma Aldrich) (200 Kunitz units/ml). Thereafter, single cell suspensions were prepared by filtering through 70 µm cell strainers (BD Biosciences, San Jose, CA). Cell viability was evaluated through Trypan blue exclusion.

### Cell sorting

A panel of antibodies (Table 5) was used to identify different DC subsets through flow cytometry. Accordingly, mouse IgG-blocked cells (Jackson ImmunoResearch, West Grove, PA) were incubated for 20 minutes at +4 °C in Brilliant Stain Buffer (BD Biosciences) in presence of the panel antibodies. Stained cells were run on a BD FACS Aria II (BD Biosciences). Briefly, doublet exclusion was performed on viable cells, which were evaluated using Fixable viability stain 520 (BD Biosciences). Leukocytes were then identified as CD45^+^ cells, out of which HLA-DR^+^ lineage^−^ (Lin) (CD3, CD14, CD56, and CD19 cells) cells were gated. Subsequently, mDCs and pDCs were identified based on expression of CD11c and CD123, respectively, and these subsets together made up the total fraction of DCs. Myeloid DCs were further subdivided into CD141^+^, CD1c^+^, and CD1c^−^CD141^−^ mDCs. The gating strategy is shown in Fig. 1A. The four identified subsets were then sorted out of malignant (n = 4) and benign (n = 4) tonsillar tissue (purity was routinely ensured to be >95%) and populations were collected in 1 ml of 100% FBS. Different sorted subset fractions (n = 32), ranging from 32 to 57035 cells, were then kept in Trizol (Life Technologies, Carlsbad, CA) and stored at −20 °C before being subjected to RNA extraction procedure.

**Table 5:** Antibody (Ab) panels applied for DC subset identification and PRR expression analysis.

Antibody	Clone	Supplier	Used for
CD45 APC-H7	2D1	BD Biosciences (CA, USA)	Sorting/marker validation
CD3 FITC	UCHT1	BD Biosciences	Marker validation
CD14 FITC	TüK4	Life Technologies (CA, USA)	Marker validation
CD56 FITC	NCAM16.2	BD Biosciences	Marker validation
CD19 Brilliant Blue515 (BB515)	HIB19	BD Biosciences	Marker validation
HLA-DR PerCp-Cy5.5	L243	BioLegend (CA, USA)	Sorting/marker validation
CD11c Brilliant Violet 421 (BV421)	B-ly6	BD Biosciences	Sorting/marker validation
CD1c APC	L161	eBioscience (CA, USA)	Sorting/marker validation
CD141 Brilliant Violet 510 (BV510)	1A4	BD Biosciences	Sorting/marker validation
CD123 PE-Cy7	6H6	BioLegend	Sorting/marker validation
CD206/MRC1 PE	3.29B1.10	Immunotech (Beckman Coulter, TX, USA)	Marker validation
CD207/Langerin PE	DCGM4	Immunotech	Marker validation
CD3 PE	UCHT1	BD Biosciences	Sorting
CD14 PE	TÜK4	Dako (Glostrup, Denmark)	Sorting
CD 19 PE	HD37	Dako	Sorting
CD56	B159	BD Biosciences	Sorting

### cRNA preparation, hybridization and upstream microarray data analysis

Total RNA amplification and labeling was performed using the Ovation Pico WTA system V2 (NuGEN, San Carlos, CA) and Encore Biotin Module Kit (NuGEN), respectively. 5 µg of the labeled RNA from the respective samples was hybridized to GeneChip Human Transcriptome Pico Assay 2.0 microarrays (Affymetrix, Santa Clara, CA) and generated signals were finally analyzed through Affymetrix GeneChip Scanner 3000 7 G platform. Microarray signal intensities were normalized through log2-transformed Robust Multi Array (RMA) algorithm on Expression Console 1.4.1.46 (Affymetrix). Two samples (one tonsillar cancer CD141^+^ mDCs and one benign tonsil CD1c^+^ mDCs) were removed from the data set due to low amount or diminished integrity of RNA.

### Transcriptional characterization of subsets in tonsillar cancer and benign tonsils

Distinctive pools of differentially expressed transcripts were identified among DC subsets in benign and malignant tonsils through applying a strategy to reduce the false discovery rate (FDR). As a brief example, significantly different transcripts exhibiting ≥1.2-fold change (FC) in expression levels (based on log2-transformed data, corresponding to ≥2-fold of non-transformed data) were identified through two-tailed t-tests comparing each subset’s transcriptome in malignant tonsils against all other three subsets’ in the same tissue. Filtered transcripts were subsequently added together to form a pool of differentially expressed transcripts among DC subsets. To remove background noise, an intensity cut-off in expression levels was applied (raw signal 100, corresponding to 6.64 in log2-transformed intensities). Two-tailed t-tests (p < 0.05) and FC filtering were performed in Omics Explorer 3.2 (Qlucore, Lund, Sweden). Thereafter, Principal Component Analysis (PCA) was performed on Qlucore Omics Explorer 3.2 to visualize the replicate similarities and their inter-subset relationships.

### Profiling of DC subsets in tonsillar cancer

To identify the transcripts selectively expressed at a higher level in individual subsets, the transcriptome of each subset in malignant tissue samples was compared to the other three subsets. Common transcripts among all the comparisons that were significantly different (one-tailed t-tests, p < 0.05) and over-expressed (FC ≥ 1.2) were considered as selectively expressed to a higher level by individual subsets. Transcripts were subsequently annotated using “Affymetrix tag ID (exon)” and transferred into MetaCore, version 6.28 build 68602, (Thomson Reuters, New York, NY). The lists of transcripts were subjected to localization gene ontology (GO) enrichment and all transcript annotations enriched in “Plasma membrane” and “Cell surface” localizations were individually reviewed. All transcripts associated with either endocytosis, cross-presentation, or signaling activities were then selected and consequently resulted in lists of markers specific for different DC subsets in malignant tonsillar tissue, which could be potential molecules for selective targeting of a specific DC subset in tonsillar cancer. The same analysis was performed to provide the selectively lower expressed transcripts by individual subsets in malignant tonsil tissue.

### Transcriptional comparison of DC subsets in malignant and benign tonsillar tissue

First, differentially expressed transcripts in DC populations in tonsillar cancer, compared to the DC counterparts in benign tonsils, were filtered using one-tailed t-test (p < 0.05) and ≥1.2-fold change in expression levels based on log2-transformed data. The lists were then imported into MetaCore and annotated using “Affymetrix tag ID (exon)” as described previously. Pathway enrichment analysis was performed on the corresponding transcripts and the top ten pathways enriched were reviewed. A q-value of 0.05 was considered as the significance cutoff to control the FDR of the enrichment analysis (pathways with FDR > 0.05 were still extracted and is marked with an asterisk in Table 2–4).

### Confirmation of marker expression using flow cytometry

To evaluate the validity of some of the selectively expressed markers, cell surface expression levels of CD206/MRC1 and CD207/Langerin were assessed using flow cytometry through a similar gating strategy to the one presented in Fig. 1. Fluorescence minus one (FMO) controls were applied and net percentage positive cells was calculated by subtracting positivity for the FMO control. Results were analyzed using FCS Express v4.0 (De Novo Software, Los Angeles, CA).

### Statistical analysis of flow cytometry data

Differences in mean percentages DCs out of CD45^+^ leukocytes, as well as in mean percentages of DC subsets out of DCs, between malignant and benign tonsillar tissue were assessed using Mann-Whitney test. Wilcoxon matched-pairs signed rank tests were used to analyze the differences between pDC and mDCs frequencies in tonsillar cancer and benign tonsils, respectively. Moreover, the frequencies of subsets expressing CD207/Langerin or CD206/MRC1 were separately compared through Wilcoxon matched-pairs signed rank test in benign and malignant tonsils. The statistical analysis was performed in GraphPad prism 7.01 (GraphPad Software, La Jolla, CA) and p-values < 0.05 were considered significant.
